# 

**Published:** 1914-03-15

**Authors:** 


### ANNOUNCEMENTS.

### Complimentary Dinner To Professor Faneuil D. Weisse.

A complimentary dinner to Faneuil D. Weisse, M.D., will be tendered by his friends in the medical and dental professions to commemorate his completion of fifty years as practitioner and teacher, at the Hotel Astor, 45th Street and Broadway, Saturday evening March 28th, 1914, at 7 o'clock.

Those desiring to attend will kindly communicate with the secretary at as early a date as possible.

Henry Sage Dunning, Secretary,
17 East 38th Street, New York City.

COMMITTEE:

W. W. Walker, Chairman,
58 West 50th Street, New York City.
H. S. Dunning, Secretary,
17 East 38th Street, New York City.
J. W. Taylor, Treasurer,
105 East 57th Street, New York City.

H. W. Gillett, W. B. Dunning,
A. R. Starr, E. Hillyer,
A. L. Swift, H. P. Gould,
R. Ottolengui, G. B. Palmer,
F. W. VanSaun.

### Notice of Removal.

The publication office of the Dental Register will remove April 1st, to the new Conrad Building, 18 and 20 W. 7th Street, Cincinnati, where the firm of Sam'l A. Crocker will have new and commodious quarters for the conduct of their business. The business has been so long at the old address that it will take time to accustom our readers to the change, but increased business required more space which could not be had in the old building, consequently the new location was selected as being more convenient to the present locations of the profession in the city.

---

### American Institute of Dental Teachers.

At the annual meeting of the Institute of Dental Pedagogics held at Buffalo, January 27th, 28th and 29th, 1914, the name of the organization was changed to American Institute of Dental Teachers. The following officers were elected for the ensuing year: President Fred W. Gethro, Chicago, Ills., Vice-President, H. M. Semans, Columbus, Ohio, Secretary-Treasurer, John F. Biddle, 517 Arch Street, N. S., Pittsburgh, Pa., Executive Board, Shirley W. Bowles, Washington, D. C., A. W. Thornton, Montreal, Canada, R. W. Bunting, Ann Arbor, Michigan.

The next annual meeting will be held at Ann Arbor, Michigan, January 28th, 29th and 30th, 1915.

J. F. Biddle, Secretary.

---

### Sixth International Dental Congress,

*London, August 3 to 8, 1914.*

Much progress has been made with the arrangements for the Congress. The British Government has issued invitations to foreign Governments and also to the self-governing Dominions of the British Empire to send official representatives to the Congress, and several delegates have already been appointed. Invitations have been issued to dental societies and organizations throughout the world, and steps have been taken to secure as Reporters or Introducers of discussions in the ten Sections of the Congress the co-operation of leading specialists and representative authorities in all branches of dental surgery.

National Committees are already at work in America,

France, and Germany &c. It is hoped that National Committees in all countries will stimulate interest in the Congress and enlist the active support of all eligible members of the profession.

The Committee of Organization has completed the following list of Sections, Officers, and Subjects for Report and Debate. Hon. Presidents of Sections, representing the various countries included in the International Dental Federation (F.D.I.), will be appointed. Those nominated by the U.S.A., France and Germany appear below. Offers of Papers on subjects other than those selected for Reports should be made as soon as possible to the Secretaries of the appropriate Sections, or to the General Secretaries, Messrs. Norman G. Bennett and H. R. F. Brooks.

---

A demonstrations Committee is being organized with Mr. T. A. Coysh as Chairman and Mr. W. F. Mellersh as Hon. Secretary.

---

The International Dental Congress Museum is intended to be representative of every Section of the Congress. Mr. A. Hopewell-Smith is Chairman of the Committee, and Mr. F. N. Doubleday is the Hon. General Secretary . Application forms and all information will be sent to alli ntending exhibitors on application at the Office of the Congress:—19, Hanover Square, W.

---

The Hon. Secretaries of Section IX would be glad to hear from gentlemen in all countries willing to give *short* demonstrations of lantern slides showing:
(*a*) Means of affording public instruction in Dental Hygiene, *e.g.*, lecture material, charts; (*b*) Photographs of School Dental Clinics or other Institutions for Public Dental Treatment. It is hoped to make this a valuable and interesting feature of the Section.

---

The Rules of the International Dental Congress provide that all ethical practitioners of dentistry possessing the qualification of the country in which they received their professional education, or of the country in which they practise, are eligible for membership.

The subscription for members of the Congress will be (7½ dollars), and for members of their families accompanying them, (3¾ dollars).

---

Messrs. T. Cook and Son, Ludgate Circus, E.C., have been appointed as official Hotel and Travel Agents for the Congress, and all applications should be made to them direct.

---

The Offices of the Congress are:—

19, Hanover Square,
London, W., to which address all communications should be sent.

---

SECTION I.—DENTAL ANATOMY, HISTOLOGY AND PHYSIOLOGY

*Subjects for Report and Debate.*

The Evolution of the Human Dentition.
Calcification.
Chemistry and Physiology of Saliva.

SECTION II.—DENTAL PATHOLOGY AND BACTERIOLOGY.

*Subjects for Report and Debate.*

The Etiology of Dental Caries.
The Etiology and Pathology of "Pyorrhoea Alveolaris."
The Pathology of the Antrum.
Pathological Conditions of the Dental Pulp.
Discussion on the British Dental Association Odontome Catalogue.

### Section III.—Dental Surgery and Therapeutics.

*Subjects for Report and Debate.*

(1) The inflammatory diseases of the gingival margin and periodontal membrane (pyorrhoea alveolaris).

(*a*) The clinical signs.
(*b*) Local Treatment.
(*c*) Treatment by ionic medication.
(*d*) Treatment by vaccines.

*Note.*—It is particularly requested that all papers should be accompanied by photographs, radiograms, &c., demonstrating the progress of cases cited and the results of treatment.

(2) The restoration of lost portions of tooth substance by inlaying.

(*a*) Inlay materials and their manipulation.
(*b*) Principles of cavity preparation.
(*c*) The cement lute.
(*d*) A comparison of inlays with "fillings."

(3) Oral Sepsis.
(*a*) Oral sepsis in relation to general disease.
(*b*) The prevention of oral sepsis.
(*c*) The treatment of oral sepsis.
(*d*) The question of crowns, bridges and dentures in relation to oral sepsis.

### SECTION IV.—DENTAL PHYSICS, CHEMISTRY, RADIOGRAPHY AND METALLURGY.

*Subjects for Report and Debate.*

The uses and advantages of X-rays as an aid to diagnosis, including the differentiation of the radiography appearances of normal and abnormal tissue.

The structural and other changes arising in connection with metals used in the mouth.

The theory and practice of pressure casting. This report will be taken at a conjoint meeting with Section V. The Reporters chosen for Section IV will deal with the theory.

### SECTION V.—DENTAL PROSTHESIS.

*Subjects for Report and Debate.*

Articulation and Articulators.

Design and Retention of Partial Dentures.

The Theory and Practice of Pressure Casting. Conjointly with Section IV. Reporters for Section V will deal with the Practice.

### SECTION VI.—ORTHODONTICS.

*Subjects for Report and Debate.*

The Unification of Terminology and Classification.

The Problem of Retention, with a view to Permanence of Result and Minimum of Danger.

Nasal Obstruction in relation to Orthodontics; in two parts, viz.: (*a*) The Effects of the Secretions of the Ductless Glands. (*b*) The effect of the Expansion of the Dental Arches, with or without the opening of the Sutures.

Root Movement.

The Relative Advantages of Fixed and Movable Appliances.

### Section VII.—Oral Surgery and Surgical Prosthesis.

*Subjects for Report and Debate.*

(1) Surgical Prosthesis of the Jaws.

(2) Late results of Cleft Palate Operations.

(3) Treatment of Dental and Dentigerous Cysts.

One morning will be devoted to each subject, the rest of the time will be devoted to papers on subjects of surgical interest in the mouth.

### Section VIII.—Anaesthesia (General and Local).

*Subjects for Report and Debate.*

Gas and Oxygen, alone, in mixture, and in sequence for the Extraction Operation.

Gas and Oxygen Analgesia for conservative operations.

Local Anaesthesia with special reference to (*a*) Methods, (*b*) Drugs, (*c*) Sphere of Usefulness, (*d*) Contra-indications and Dangers.

### Section IX.—Oral Hygiene, Public Instruction, and Public Dental Services.

*Subjects for Report and Debate.*

The Effects of Dental Treatment on National Health and Physique.

Prophylaxis at different ages.

Lantern demonstration of slides showing (*a*) Means of affording public instruction in Dental Hygiene, *e.g.*, lecture material, charts, &c.; (*b*) Photographs of School Dental Clinics, Institutions for Public Dental Service for Adults, or other Institutions in which public dental treatment is being carried out. To be contributed to by all countries willing to send representatives. It is intended that many dentists should exhibit slides showing the working of institutions, or of public instruction, and very briefly describe them.

### Section X.—Dental Education.

*Subjects for Report and Debate.*

The Teaching of Bacteriology for Dental Students, with special reference to (*a*) the Method of teaching; (*b*) the Extent of the teaching.

A Practical Synopsis of Medical and Surgical Teaching for Dental Students.

First Principles in Practical Teaching.

Methods of teaching Orthodontics to Dental Students.

